# Sports participation and health-related quality of life in children: results of a cross-sectional study

**DOI:** 10.1186/s12955-019-1124-y

**Published:** 2019-04-15

**Authors:** Janet Moeijes, Jooske T. van Busschbach, Thomas H. Wieringa, Jordy Kone, Ruud J. Bosscher, Jos W. R. Twisk

**Affiliations:** 1 Department of Human Movement and Education, Windesheim University of Applied Sciences, Campus 2-6, Zwolle, 8017CA The Netherlands; 2 Department of Epidemiology and Biostatistics, Amsterdam University Medical Centers (AMC and VUMC), Amsterdam Public Health research institute, Van der Boechorststraat 7, 1081BT Amsterdam, The Netherlands; 3 University of Groningen, University Medical Center Groningen, University Center for Psychiatry, P.O. Box 30001, 9700 RB Groningen, The Netherlands; 4 Department of Medical Psychology, Amsterdam University Medical Centers (AMC and VUMC), Amsterdam Public Health research institute, Van der Boechorststraat 7, 1081 BT Amsterdam, the Netherlands; 5 School of Health Care Studies, Hanze University of Applied Sciences Groningen, P.O. Box 30030, 9700 RM Groningen, The Netherlands; 6 Department of Health and Welfare, Windesheim University of Applied Sciences, Campus 2-6, Zwolle, 8017CA The Netherlands

**Keywords:** Membership sports club, Frequency of sports participation, Individual sports, Team sports, Indoor sports, Outdoor sports, Health-related quality of life, KIDSCREEN-52

## Abstract

**Background:**

In children physical activity has been shown to be associated with health-related quality of life (HRQoL). This study further explores this association for specific characteristics of sports participation, namely membership of a sports club, frequency of sports participation, performing individual versus team sports, performing indoor versus outdoor sports, while differentiating between specific dimensions in the physical, psychological and social domain of HRQoL.

**Methods:**

Cross-sectional data were collected from Dutch primary school children aged 10 to 12 years. They completed the Movement and Sports Monitor Questionnaire Youth aged 8 to 12 years (MSMQ) and the KIDSCREEN-52, an HRQoL questionnaire for children and adolescents. The data were examined using linear multilevel analyses because of the clustering of children in schools.

**Results:**

The questionnaires were completed by 1876 children (response rate 81.3%). Membership of a sports club, moderate or high frequency of sports participation, and performing outdoor sports were all significantly associated with better HRQoL. These associations were largely found in the physical domain of HRQoL, to a lesser degree in the social domain, and to a limited extent in the psychological domain.

**Conclusion:**

The association between sports participation and HRQoL in children depends on both characteristics of sports participation and the domain of life that is concerned. These differences offer starting points for developing tailor-made sports programs for children.

**Electronic supplementary material:**

The online version of this article (10.1186/s12955-019-1124-y) contains supplementary material, which is available to authorized users.

## Background

The last 50 years witnessed a shift in the nature, perception, and treatment of children’s health and illness [1, 2]. Because of the progress in medical care, pediatric medicine gradually focused less on diagnosis and treatment of infectious diseases and more on prevention and promotion of conditions that are favourable for physical health [1, 3]. Moreover, there has been an increase in the observed prevalence of mental health problems in children [4]. In this context, there is a need for outcome measures that do not primarily reflect the biomedical model but focus more on the relationship between physical and mental health [3]. Such an outcome measure is quality of life (QoL). QoL is considered essential for the evaluation of measures in the field of prevention, treatment, and rehabilitation [5–7].

QoL is a multidimensional concept [8, 9]. It can be defined as subjectively perceived well-being and satisfaction with the physical, emotional, mental, social, and behavioural components of functioning [10, 11]. Health-related QoL (HRQoL) is a subset of quality of life [12, 13] focusing on the three central elements in the World Health Organization’s definition [14] of health, namely physical, mental, and social well-being [8, 13, 15].

Several studies in adults and adolescents suggest sports participation to be associated with a more favourable HRQoL. Being physically active in a socially engaged manner by participating in sports activities seems to improve HRQoL in adults and adolescents [16–18].

In the pediatric literature, a number of studies link a broad spectrum of physical activities to HRQoL without explicitly distinguishing sports participation as a separate component of a child’s physical activities [12, 19–25]. Only a few studies paid specific attention to a child’s sports participation and HRQoL reporting a positive relationship between both [26, 27]. To gain more insight in this association, Coalter [28] advocates that studies on the intended benefits of sports participation should not be of a general character but need to focus on specific characteristics of sports participation. To the best of our knowledge, there is only one such study that examined the relationships between one or more specific characteristics of sports participation and HRQoL in children. Vella, et al. [27] observed that children performing team sports or performing team sports as well as individual sports had a more favourable HRQoL compared to children performing only individual sports and children not participating in sports. Apart from these results, little is known about the associations between various characteristics of sports participation and HRQoL in children.

In line with Vella, et al. [27], the current study aimed to investigate the associations between four specific characteristics of sports participation and the physical, psychological and social domain of HRQoL in children. The study focused on participation in sports club activities, which is the dominant form of children’s sports participation in the Netherlands [29]. The characteristics of sports participation included are membership of a sports club, frequency of sports participation, performing individual versus team sports, and performing indoor versus outdoor sports.

## Methods

### Participants

In several waves over a period of 3 years (November 2011 to April 2014) fourth and fifth–grade children from Dutch primary schools in both urban and rural regions with a broad range of SES levels in the Netherlands were invited to participate in this cross-sectional study. The 73 schools that were willing to participate in the study (response rate 63%) were geographically spread across the Netherlands.

This resulted in a sample of in total 2308 children (response rate 72%) that is comparable with the general Dutch population of primary school children where neighbourhood socioeconomic status (SES) is concerned (7.8% vs 11.6% low SES and 12.2% vs 16.6% high SES) [30, 31], the proportion of overweight children (based on body mass index (BMI) 15.0% vs 12.5%) [32] and, membership of a sports club (85.3% vs 78.0% member) [31, 33].

### Design and procedures

School directors were first approached by phone and email. If a school director showed interest in the study, an email with detailed information was sent, and he or she was again contacted by phone 1 week later. After permission was given by the school director, parents or guardians of the children received written information about the aim, nature, and practice of the research. Written informed consent from a parent or guardian was a prerequisite to participate in this study.

The majority of schools (44) were involved in the study only once. A number of schools participated with different classes in two waves (21) or in three (8).

During an initial meeting, children were presented a booklet containing questionnaires that had to be completed individually in the classroom. If assistance was needed, children could consult their classroom teacher. One week later, anthropometric data were obtained during school time. Questionnaires and other procedures were tested in advance in a small target sample of fourth and fifth-grade children in three primary schools.

### Measures

#### Health-related quality of life (HRQoL)

HRQoL was measured using the KIDSCREEN-52, a self–report generic measure of HRQoL in children and adolescents aged between 8 and 18 [8, 34]. The original English version of the questionnaire was translated into a Dutch version, using a standardized methodology based on international cross-cultural translation guidelines [35, 36].

The KIDSCREEN-52 comprises 52 items covering ten dimensions in three domains. The ‘physical well-being’ dimension relates to the physical domain and includes 5 items. The psychological domain comprises the dimensions ‘psychological well-being’ (6 items), ‘moods and emotions’ (7 items), and ‘self-perception’ (5 items). The social domain relates to the dimensions ‘autonomy’ (5 items), ‘parent relations and home life’ (6 items), ‘social support and peers’ (6 items), ‘school environment’ (6 items), ‘social acceptance (bullying)’ (3 items), and ‘financial resources’ (3 items). All items are rated on a 5-point Likert scale (ranging from “never” to “always” or from “not at all” to “extremely”). After recoding some of the items, a higher score indicates a more favourable HRQoL.

The KIDSCREEN-52 has a satisfactory internal consistency with Cronbach’s alphas for the ten dimensions ranging from 0.77 to 0.89 [8, 10]. The test-retest reliability is also satisfactory with intraclass correlation coefficients (ICCs) ranging from 0.56 to 0.77 [8, 10]. The questionnaire shows good results in terms of convergent, known groups’ and criterion validity [10].

#### Sports participation

Sports participation was assessed with the Movement and Sports Monitor Questionnaire – Youth Aged 8–12 Years (MSMQ) [37]. This questionnaire contains, among others, items about membership of a sports club and frequency of sports participation. An item concerning the kind of sport(s) in which the child participated was added. The validation of the items on sports participation is described elsewhere [38].

Sports participation was characterized by four variables: whether or not a child is a member of a sports club; frequency of participation in the sports club, with scores divided into tertiles that distinguish between low sports-active (0.50–2.20 times per week), moderate sports-active (2.25–3.00 times per week), and high sports-active children (3.02–14.00 times per week); performing individual versus team sports; and performing indoor versus outdoor sports. Team sports were defined as sports in which two or more persons work together as allies to get an optimal joint result. Individual sports were defined as sports in which one single person acts on his own, striving for an optimal individual result [39].

#### Covariates

We treated gender (dichotomous), body mass index (BMI; continuous), age (continuous), neighbourhood socioeconomic status (SES; continuous), and household composition (dichotomous) as potential confounders. According to literature, these factors are associated with HRQoL [6, 23, 40, 41]. For the analyses of the relationships between the independent variables ‘performing individual versus team sports’ and ‘performing indoor versus outdoor sports’ on the one hand and the ten dimensions of HRQoL on the other, we also added frequency of sports participation (continuous) as a potential confounder.

Parents or guardians reported the gender and day of birth. Height and weight were assessed by researchers visiting the schools, using validated scales. BMI was calculated by dividing weight by height squared (kg/m^2^) [42]. Whether a child lived in a two-parent family or another type of household, for instance a one-parent family, was operationalised in the variable ‘household composition’ [43]. SES of the child’s parents or guardians was based on SES scores for each postal code (i.e. neighbourhood) derived from the National Dutch Social and Cultural Planning Office in the year 2014 [44]. In the Netherlands, this neighbourhood SES score is derived from a number of characteristics of neighbourhood residents, such as education level, income and labour market position. It is a standardized score with an average of 0 and a standard deviation of 1 [44].

#### Statistical analyses

As described in the KIDSCREEN manual, Rasch scores were calculated for each dimension of HRQol and were transformed into T-values with a mean of 50 and a standard deviation of 10 [8, 45].

Children without complete information on the sports participation questionnaire MSMQ, BMI, SES, or household composition were excluded from the analyses. In case of missing values on the KIDSCREEN-52, the score on a dimension (except the 3-item dimensions ‘social acceptance (bullying)’ and ‘financial resources’) was only calculated if not more than one item within the dimension was left unanswered [8]. In order to examine the impact of missing data on the sports characteristics and covariates, sensitivity analyses were performed using t-tests and chi-square tests. SPSS (version 24, IBM, New York, United States) was used for the analyses described above.

The dataset had a hierarchical structure, due to within-school clustering: children (level 1), clustered in schools (level 2). Multilevel models were used to take into account the hierarchical clustering. For the outcome variables (i.e., the ten dimensions of the KIDSCREEN-52) multilevel models included a random intercept for the schools.

We chose to analyse associations between each of the characteristics of sports participation and each of the ten outcome variables separately using linear multilevel regression analyses. These analyses were performed in two steps: univariate analyses uncorrected for potential confounding and multivariate analyses corrected for potential confounding. Before the two steps regression analyses, we checked for the potential presence of multicollinearity. Almost all potential confounders covaried with characteristics of sports participation slightly. Frequency of sports participation, however, covaried with performing individual versus team sports and performing indoor versus outdoor sports moderately. Because of the exploratory nature of the study, no adjustment for multiple testing was performed [46, 47].

STATA (version 13.1; Stata Corporations, College Station, Texas) was used for these analyses.

A *p*-value for statistical significance was set at 0.05 for all analyses.

## Results

From the 2308 children who participated in the study, 1876 children (81.3%), completed questionnaires and anthropometric measurements. Data from 432 children (18.7%) were not included in the analyses due to missing data on the MSMQ, KIDSCREEN-52, BMI, SES or household composition. No significant differences between children with complete data and dropouts were observed for gender, age, BMI, neighbourhood SES, household composition, the characteristics of sports participation and almost all ten HRQoL dimensions. Children of whom information was not used because of missing data did show worse scores on the dimension ‘financial resources’ (*p* = 0.03). Table 1 shows descriptive information about the sample under study.

**Table 1:** General characteristics of the sample (*n* = 1876)

Demographic variables	Specification	Frequency (%)	Mean ± SD/Median [range]
Gender	Boys	890 (47.4)	
	Girls	986 (52.6)	
Age			11.5 ± 0.70
BMI			18.06 ± 2.72
SES			0.24 ± 1.07
Two parents household	No	346 (18.4)	
	Yes	1530 (81.6)	
Membership sports club	No	273 (14.6)	
	Yes	1603 (85.4)	
Weekly frequency of sports participation			3.03 ± 1.46
	Low sports-active	535 (33.4)	2 [0.50–2.20]
	Moderate sports active	532 (33.2)	3 [2.25–3.00]
	High sports active	536 (33.4)	4 [3.02–14.00]
Individual versus team sports	Individual sports	410 (25.6)	
	Team sports	888 (55.4)	
	Individual sports as well as team sports	305 (19.0)	
Indoor versus outdoor sports	Indoor sports	559 (35.9)	
	Outdoor sports	824 (51.4)	
	Indoor sports as well as outdoor sports	220 (13.7)	
KIDSCREEN-52	Physical domain		
	Physical wellbeing		55.58 ± 9.71
	Psychological domain		
	Psychological wellbeing		54.86 ± 9.71
	Moods and emotions		51.53 ± 10.37
	Self-perception		54.44 ± 9.40
	Social domain		
	Autonomy		54.92 ± 8.76
	Parent relation and home life		56.00 ± 8.55
	Social support and peers		53.57 ± 8.96
	School environment		56.19 ± 8.98
	Social acceptance (bullying)		49.07 ± 10.40
	Financial resources		54.39 ± 9.12

Results of the univariate and multivariate analyses regarding the associations between the four characteristics of sports participation on the one hand and the ten dimensions of HRQoL on the other are presented in Tables 2, 3, 4 and 5. To create the best overview, we structured the results and tables along the lines of the four characteristics of sports participation.

**Table 2:** Associations between membership of a sports club and HRQOL-dimensions (*n* = 1876)

		Crude analyses			Adjusted analyses^a^		
		B^b^	p^c^	95% CI	B^b^	p^c^	95% CI
Physical domain							
Physical wellbeing	Non-member	Reference group					
	Member	4.78	**< 0.001**	3.54; 6.01	4.26	**< 0.001**	3.04; 5.48
Psychological domain							
Psychological wellbeing	Non-member	Reference group					
	Member	0.96	0.11	−0.20; 2.13	0.88	0.14	−0.29; 2.05
Moods and emotions	Non-member	Reference group					
	Member	0.95	0.17	− 0.39; 2.29	0.87	0.21	−0.48; 2.22
Self-perception	Non-member	Reference group					
	Member	1.65	**0.01**	0.44; 2.87	1.26	**0.04**	0.07; 2.44
Social domain							
Autonomy	Non-member	Reference group					
	Member	1.67	**0.004**	0.53; 2.80	1.74	**0.003**	0.59; 2.88
Parents relations and home life	Non-member	Reference group					
	Member	1.25	**0.03**	0.15; 2.36	1.38	**0.02**	0.27; 2.48
Social support and peers	Non-member	Reference group					
	Member	1.64	**0.01**	0.48; 2.80	1.67	**0.01**	0.50; 2.84
Social acceptance (bullying)	Non-member	Reference group					
	Member	1.61	**0.02**	0.26; 2.95	1.56	**0.03**	0.20; 2.92
School environment	Non-member	Reference group					
	Member	0.59	0.32	−0.57; 1.74	0.78	0.18	− 0.37; 1.94
Financial resources	Non-member	Reference group					
	Member	2.47	**< 0.001**	1.30; 3.65	2.47	**< 0.001**	1.28; 3.65

^a^Adjusted for gender, age, BMI, SES, and household composition; ^b^Unstandardized regression coefficient; ^c^*p*-values in bold indicate statistical significance (*p* < 0.05)

**Table 3:** Associations between frequency of sports participation and HRQOL-dimensions for sports club members (*n* = 1603)

		Crude analyses			Adjusted analyses^a^		
		B^b^	p^c^	95% CI	B^b^	p^c^	95% CI
Physical domain							
Physical wellbeing	High sports-active	Reference group					
	Moderate sports-active	−0.79	0.17	−1.92; 0.34	−0.90	0.11	−2.01; 0.21
	Low sports-active	−3.91	**< 0.001**	−5.06; − 2.77	−3.52	**< 0.001**	−4.65; − 2.39
Psychological domain							
Psychological wellbeing	High sports-active	Reference group					
	Moderate sports-active	−0.54	0.32	−1.61; 0.53	− 0.58	0.28	− 1.64; 0.48
	Low sports-active	−1.67	**0.003**	−2.74; −0.59	−1.48	**0.01**	−2.57; −0.40
Moods and emotions	High sports-active	Reference group					
	Moderate sports-active	0.56	0.37	−0.68; 1.80	0.47	0.45	−0.76; 1.71
	Low sports-active	−0.34	0.60	−1.59; 0.92	−0.16	0.81	−1.42; 1.10
Self-perception	High sports-active	Reference group					
	Moderate sports-active	0.62	0.28	−0.50; 1.74	0.36	0.52	−0.73; 1.45
	Low sports-active	−0.21	0.72	−1.34; 0.92	0.28	0.63	−0.83; 1.38
Social domain							
Autonomy	High sports-active	Reference group					
	Moderate sports-active	0.05	0.92	−0.99; 1.10	−0.06	0.92	−1.10; 0.99
	Low sports-active	−0.21	0.69	−1.26; 0.84	0.01	0.99	−1.05; 1.06
Parent relations and home life	High sports-active	Reference group					
	Moderate sports-active	−0.73	0.16	−1.74; 0.28	−0.78	0.13	−1.78; 0.22
	Low sports-active	−1.57	**0.003**	−2.60; −0.55	−1.54	**0.003**	−2.57; −0.52
Social support and peers	High sports-active	Reference group					
	Moderate sports-active	−0.82	0.14	−1.89; 0.25	−0.75	0.17	−1.82; 0.32
	Low sports-active	−0.85	0.12	−1.94; 0.22	−0.77	0.16	−1.86; 0.31
Social acceptance (bullying)	High sports-active	Reference group					
	Moderate sports-active	0.89	0.16	−0.34; 2.11	1.02	0.10	−0.21; 2.24
	Low sports-active	−0.32	0.62	−1.56; 0.92	−0.45	0.49	−1.70; 0.81
School environment	High sports-active	Reference group					
	Moderate sports-active	−0.61	0.26	−1.66; 0.44	−0.37	0.49	−1.41; 0.67
	Low sports-active	−0.70	0.20	−1.77; 0.37	−1.15	**0.03**	−2.22; − 0.09
Financial resources	High sports-active	Reference group					
	Moderate sports-active	−0.68	0.21	−1.72; 0.35	−0.59	0.26	−1.62; 0.44
	Low sports-active	−1.33	**0.01**	−2.38; −0.29	−1.24	**0.02**	−2.29; − 0.18

^a^Adjusted for gender, age, BMI, SES, and household composition; ^b^Unstandardized regression coefficient; ^c^*p*-values in bold indicate statistical significance (*p* < 0.05)

**Table 4:** Associations between performing individual versus team sports and HRQOL-dimensions for sports club members (*n* = 1603)

		Adjusted analyses^a^			Adjusted analyses^b^		
		B^c^	p	95% CI	B^c^	p	95% CI
Physical domain							
Physical wellbeing	Individual sports	Reference group					
	Team sports	0.50	0.37	−0.60; 1.61	− 0.06	0.92	−1.16; 1.05
	Individual as well as team sports	1.76	0.13	0.37; 3.14	− 0.56	0.48	−2.09; 0.98
Psychological domain							
Psychological wellbeing	Individual sports	Reference group					
	Team sports	0.67	0.21	−0.38; 1.72	0.46	0.39	−0.60; 1.52
	Individual as well as team sports	0.81	0.23	−0.51; 2.13	−0.08	0.93	−1.54; 1.41
Moods and emotions	Individual sports	Reference group					
	Team sports	−0.20	0.75	−1.41; 1.02	−0.16	0.80	−1.39; 1.07
	Individual as well as team sports	−0.46	0.56	−1.99; 1.07	−0.30	0.73	−2.02; 1.41
Self-perception	Individual sports	Reference group					
	Team sports	−0.61	0.26	−1.68; 0.46	− 0.61	0.27	− 1.69; 0.47
	Individual as well as team sports	0.37	0.59	−0.98; 1.71	0.39	0.61	−1.11; 1.90
Social domain							
Autonomy	Individual sports	Reference group					
	Team sports	0.23	0.66	−0.79; 1.26	0.21	0.70	−0.83; 1.24
	Individual as well as team sports	−0.09	0.89	−1.38; 1.19	−0.21	0.78	−1.65; 1.24
Parents and homelife	Individual sports	Reference group					
	Team sports	0.53	0.30	−0.46; 1.52	0.30	0.55	−0.69; 1.30
	Individual as well as team sports	0.44	0.49	−0.80; 1.69	−0.48	0.50	−1.87; 0.92
Social support and peers	Individual sports	Reference group					
	Team sports	0.80	0.13	−0.25; 1.86	0.65	0.23	−0.41; 1.72
	Individual as well as team sports	1.08	0.11	−0.24; 2.40	0.44	0.56	−1.04; 1.93
Social acceptance (bullying)	Individual sports	Reference group					
	Team sports	0.98	0.11	−0.23; 2.19	0.99	0.11	−0.24; 2.21
	Individual as well as team sports	0.89	0.25	−0.63; 2.41	0.91	0.30	−0.79; 2.62
School environment	Individual sports	Reference group					
	Team sports	−0.59	0.26	−1.62; 0.44	−0.78	0.14	− 1.82; 0.26
	Individual as well as team sports	0.41	0.54	−0.89; 1.70	−0.39	0.60	−1.84; 1.06
Financial resources	Individual sports	Reference group					
	Team sports	0.58	0.26	−0.43; 1.60	0.35	0.51	−0.68; 1.37
	Individual as well as team sports	0.81	0.21	−0.47; 2.09	−0.18	0.81	−1.61; 1.25

^a^Adjusted for gender, age, BMI, SES, and household composition; ^b^Adjusted for gender, age, BMI, SES, household composition and frequency of sports participation; ^c^Unstandardized regression coefficient

**Table 5:** Associations between performing indoor versus outdoor sports and HRQOL-dimensions for sports club members (*n* = 1603)

		Adjusted analyses^a^			Adjusted analyses^b^		
		B^c^	p^d^	95% CI	B^c^	p^d^	95% CI
Physical domain							
Physical wellbeing	Indoor sports	Reference group					
	Outdoor sports	1.81	**0.001**	0.73; 2.88	0.97	0.08	−0.13; 2.06
	Indoor versus outdoor sports	1.13	0.13	−0.32; 2.59	− 1.02	0.20	−2.59; 0.55
Psychological domain							
Psychological wellbeing	Indoor sports	Reference group					
	Outdoor sports	0.89	0.09	−0.13; 1.92	0.54	0.31	−0.51; 1.59
	Indoor versus outdoor sports	−0.11	0.88	−1.49; 1.27	−1.02	0.19	−2.53; 0.49
Moods and emotions	Indoor sports	Reference group					
	Outdoor sports	1.31	**0.03**	0.12; 2.50	1.37	**0.03**	0.15; 2.59
	Indoor versus outdoor sports	−0.71	0.39	−2.31; 0.90	−0.55	0.54	−2.30; 1.20
Self-perception	Indoor sports	Reference group					
	Outdoor sports	0.98	0.07	−0.06; 2.03	0.97	0.08	−0.11; 2.04
	Indoor versus outdoor sports	0.32	0.66	−1.10; 1.73	0.28	0.73	−1.27; 1.82
Social domain							
Autonomy	Indoor sports	Reference group					
	Outdoor sports	0.77	0.13	−0.23; 1.77	0.70	0.18	−0.32; 1.73
	Indoor versus outdoor sports	−0.83	0.23	−2.18; 0.52	−1.01	0.18	−2.48; 0.47
Parents and home life	Indoor sports	Reference group					
	Outdoor sports	0.50	0.31	−0.47; 1.47	0.16	0.76	−0.83; 1.15
	Indoor versus outdoor sports	−0.21	0.75	−1.52; 1.10	−1.10	0.13	−2.53; 0.32
Social support and peers	Indoor sports	Reference group					
	Outdoor sports	−0.09	0.86	−1.12; 0.94	−0.42	0.44	−1.47; 0.64
	Indoor versus outdoor sports	−0.40	0.57	−1.80; 0.99	−1.25	0.11	−2.76; 0.27
Social acceptance (bullying)	Indoor sports	Reference group					
	Outdoor sports	2.15	**< 0.001**	0.97; 3.33	2.16	**< 0.001**	0.95; 3.38
	Indoor versus outdoor sports	0.44	0.59	−1.15; 2.03	0.47	0.60	−1.27; 2.20
School environment	Indoor sports	Reference group					
	Outdoor sports	−0.85	0.10	−1.86; 0.16	−1.21	**0.02**	−2.24; −0.17
	Indoor versus outdoor sports	−0.19	0.78	−1.55; 1.17	−1.12	0.14	−2.60; 0.36
Financial resources	Indoor sports	Reference group					
	Outdoor sports	0.93	0.07	−0.06; 1.93	0.58	0.27	−0.44; 1.60
	Indoor versus outdoor sports	0.41	0.55	−0.94; 1.75	−0.51	0.49	−1.97; 0.95

^a^Adjusted for gender, age, BMI, SES, and household composition; ^b^Adjusted for gender, age, BMI, SES, household composition and frequency of sports participation; ^c^Unstandardized regression coefficient; ^d^*p*-values in bold indicate statistical significance (*p* < 0.05)

Table 2 shows that membership of a sports club was significantly associated with better HRQoL. This is most prominent in the physical and social domain, and to a lesser extent in the psychological domain.

In children who were a member of a sports club, high sports-active children showed substantially better HRQoL than low sports-active children. As was the case for membership, the associations were most strong in the physical and social domain (Table 3). No significant difference in this respect was observed between high-active and moderate sports-active children. There were also no significant differences found between moderate sports-active and low sports-active children, except for the dimensions of ‘physical wellbeing’ and ‘social acceptance (bullying)’, with better HRQoL for moderate sports-active children.

As is apparent from Table 4, no significant differences in HRQoL were observed between children performing individual sports, children performing team sports, and children performing both individual and team sports.

As shown in Table 5, performing outdoor sports was significantly associated with more favourable HRQoL in the ‘moods and emotions’ dimension compared with performing indoor sports. Performing outdoor sports was also significantly associated with favourable scores on ‘social acceptance (bullying)’ and unfavourable scores on ‘school environment’ compared with performing indoor sports. No significant associations were found between performing indoor sports versus outdoor sports in the other dimensions. Additional file 1 contains the results of the crude analyses corresponding with the results of the adjusted analyses presented in Tables 4 and 5.

## Discussion

This study aimed to explore associations between four characteristics of sports participation and HRQoL in primary schoolchildren in the fourth and fifth grade. Membership of a sports club, moderate or high frequency of sports participation, and performing outdoor sports (compared to indoor sports) were characteristics of sports participation that were related to a more favourable HRQoL. These associations were largely found in the physical domain, to a lesser degree in the social domain, and to a limited extent in the psychological domain of HRQoL.

### Membership of a sports club

The observed associations between being a member of a sports club and HRQoL are in line with those of previous studies for adults [17, 18], adolescents [16, 48, 49], and children [26, 27]. These studies also reported a positive relationship between, amongst others, being a member of a sports club and one or more specific dimensions of HRQoL.

Concerning the physical domain of HRQoL, the explanation for this association may be that members of a sports club are in general more active and thus have a better physical condition [50, 51], which in turn results in higher physical well-being [12, 52]. In the social domain, the positive associations of membership of a sports club with almost all HRQoL dimensions suggest that organised sports activities facilitate more positive social experiences [20, 23], leading to better HRQoL. Moreover, being a member of a sports club might result in feelings of inclusion, in experiencing social support, and so offer ways to resist bullying or to compensate for its adverse effect [16]. Contrary to some scholars who report negative effects of sports participation [53, 54], our research findings are, therefore, positive in the social domain. However, one could argue that children who have difficulties to engage in positive behaviours towards peers and stand at risk of being bullied might refrain from becoming a member of a sports club [55]. In the psychological domain, the positive association of being a member of a sports club with ‘self-perception’ might be explained by the child’s experience of being more competent [56] and the opportunity to gain success experiences. Sports participation also offers the opportunity of developing motor skills, which contributes to a positive sports self-perception as an important element of general self-perception [57–59].

### Frequency of sports participation

Within the group of children who joined a sports club, moderate and high frequency of sports participation were associated with more favourable HRQoL for one or more dimensions in each of the three HRQoL domains. These findings are in line with those of other studies performed in adults [17], adolescents [60, 61] and children [20, 24, 27, 62], which reported positive HRQoL outcomes of frequent physical or sports activities.

For the physical domain, the observed associations with ‘physical wellbeing’ might be explained by the ‘feel-good’ effect that sports participation entails. The ‘feel-good’ effect refers to enhanced feelings of energy, vigour, pleasant mood, and joy after sports participation at an at least moderate activity level [63]. In the social domain, frequency of sports participation was positively associated with a substantial number of HRQoL dimensions. There was, however, an ambiguous picture with respect to ‘social acceptance (bullying)’. On the one hand, moderate sports-active children suffered less from bullying than low sports-active children. Due to their sports activities, moderate sports-active children might have developed more social skills and greater physical strength to defend themselves against bullies compared to low sports-active children [64]. On the other hand, high sports-active children did not show better scores on ‘social acceptance (bullying)’ compared to low and moderate sports-active children. Vertommen, et al. [65] suggest that high sports-active children are more likely to practice their sports activities in an environment that generates competitive feelings in children and possibly reinforces aggressive behaviour. In such an environment, that is hard to escape from because of the sacrifices made and the potential benefits, children will probably have to tolerate more bullying behaviours of rivals [66].

In the psychological domain, the positive association with ‘psychological well-being’ might be attributed to the fact that high sports-active children, who spend several days a week a certain time at a sports club, fulfil their psychological needs for autonomy, competence, and relatedness [67].

### Performing team versus individual sports

The only study that examined performing individual versus team sports to HRQoL in primary school children was performed by Vella et al. [27]. They found that performing team sports was associated with better HRQoL. The present study showed no such association. An explanation might be that the distinction between individual and team sports is not that straightforward. Children participating in individual sports usually train together in groups just like children in team sports and, therefore, largely share the same group processes as team athletes [68]. The positive effects of being a member of a group on HRQoL [40] might occur not only in children performing team sports but also in children performing individual sports.

### Performing indoor versus outdoor sports

The current research findings relating HRQoL to indoor versus outdoor sports are to a large extent consistent with those of previous studies in adults [69], adolescents [70], and children [71].

For the physical domain, we did not find a significant association between performing indoor versus outdoor sports and ‘physical well-being’. Both indoor and outdoor athletes seem to benefit equally in terms of physical well-being in contrast to having a more sedentary or sports-inactive lifestyle [71]. Furthermore, there are indications that the kind of natural environment in which physical activities takes place has no clear impact on someone’s ‘physical well-being’ [69]. Regarding the psychological domain, however, it was observed that children participating in outdoor sports showed better ‘moods and emotions’ than indoor sporting peers. This may be because playing in the open air, the surrounding greenness, and performing physical activities in nature are conducive to a positive mood and less depressing symptoms [70–74]. Concerning the social domain, the more favourable social acceptance (less bullying) reported by outdoor sporting children might be caused by the fact that outdoor activities provide children with more opportunities to gain physical strength and endurance, for instance, due to changing weather conditions in the open air (e.g., rain, wind power, and sunshine). Their higher physical strength and endurance support children when resisting the bully [75]. A possible explanation for the higher dissatisfaction regarding school environment in children doing outdoor sporting activities is that these children are probably more interested in the outdoor environment and less fond of intramural school activities [76, 77].

### Strengths and limitations

As stated, except for Vella, et al. [27], we know of no other study that examined associations between various characteristics of sports participation and several dimensions of HRQoL in children. The present study fills this gap by investigating a representative sample of a large number of primary school children which favours the generalizability of the findings. The observed associations between membership of a sports club, frequency of sports participation, and the type of sports participation on the one hand, and HRQoL on the other, thus constitute a first step towards better understanding and development of sports programs for children. There is some evidence that the observed relationship between sports participation and HRQoL in children is also present in low-income countries. Van Hout, et al. [48] and Salvini, et al. [78] found this positive relationship in South African children and adolescents.

Some limitations should, however, be considered. First, the characteristics of sports participation were determined using the self-report questionnaire MSMQ, without using additional objective measures such as accelerometers, pedometers and heart rate monitors. As is common practice in large-scale cross-sectional studies, only a self-report questionnaire was chosen for reasons of time-saving and cost efficiency. Future research should preferably make use of a combination of self-report questionnaires and data of accelerometers. This way, additional characteristics of sports participation such as duration and intensity, could also be taken into account.

Second, there may be a multitesting problem due to the many statistical analyses that include the risk of Type I errors. However, we did not correct for multitesting since in an exploratory study, in which many variables are involved, such a correction seems to be less necessary. In addition, by using multiple testing adjustment, potentially meaningful findings could be missed [46, 47].

Third, the cross-sectional design of the study precludes the investigation of causal relationships and neglects potential bidirectional effects [61, 79, 80]. Qualitative or mixed methods design studies are desirable to gain insight into the underlying working mechanisms.

## Conclusions

To the best of the authors’ knowledge, this is one of the first studies examining associations between various characteristics of sports participation and several dimensions of HRQoL in primary school children. Membership of a sports club, moderate or high frequency of sports participation, and performing outdoor sports (instead of indoor sports) appeared to be significantly associated with a more favourable HRQoL. The distinction between individual and team sports did not appear to be relevant. The associations were largely found in the physical domain, to a lesser degree in the social domain, and to a limited extent in the psychological domain of HRQoL. The research findings may be useful for childhood health-related interventions. Children should be encouraged to become member of a sports club and to participate in organised sports activities moderate or high frequently, preferably in an outdoor setting. Future research should preferably have a qualitative or mixed methods design.

## Additional file

Additional file 1: **Table S4.** Crude and adjusted analyses of the associations between performing individual versus team sports and HRQOL-dimensions for sports club members (*n* = 1603). **Table S5.** Crude and adjusted analyses of the associations between performing indoor versus outdoor sports and HRQOL-dimensions for sports club members (*n* = 1603). (DOCX 48 KB)

## Abbreviations

BMI: Body Mass Index
HRQoL: Health Related Quality of Life
KIDSCREEN-52: A self–report generic measure of HRQoL in children and adolescents aged between 8 and 18
MSMQ: Movement and Sports Monitor Questionnaire -Youth aged 8–12 years
QoL: Quality of Life
SES: Socioeconomic Status
