# Supplementary material for: Sports participation and health-related quality of life in children: results of a cross-sectional study
Source: Health Qual Life Outcomes. 2019 Apr 15;17:64. doi: 10.1186/s12955-019-1124-y (PMC6466683; doi:10.1186/s12955-019-1124-y)
Supplement: Supplementary file 1 — Table S4. Crude and adjusted analyses of the associations between performing individual versus team sports and HRQOL-dimensions for sports club members (n = 1603). Table S5. Crude and adjusted analyses of the associations between performing indoor versus outdoor sports and HRQOL-dimensions for sports club members (n = 1603). (DOCX 48 KB) [file 12955_2019_1124_MOESM1_ESM.docx]

Table S4. Crude and adjusted analyses of the associations between performing individual versus team sports and HRQOL-dimensions for sports club members (n=1,603)

|  |  | | Crude analyses | | | Adjusted analyses^a^ | | | Adjusted analyses^b^ | | |
| --- | --- | --- | --- | --- | --- | --- | --- | --- | --- | --- | --- |
|  |  | | B^c^ | p^d^ | 95%CI | B^c^ | p^d^ | 95%CI | B^c^ | p^d^ | 95%CI |
| **Physical domain** | |  |  | | |  |  |  |  |  |  |
| Physical wellbeing | Individual sports | | Reference group | | |  |  |  |  |  |  |
|  | Team sports | | 0.90 | 0.12 | -0.22; 2.02 | 0.50 | 0.37 | -0.60; 1.61 | -0.06 | 0.92 | -1.16; 1.05 |
|  | Individual as well as team sports | | 1.65 | **0.02** | 0.23; 3.08 | 1.76 | 0.13 | 0.37; 3.14 | -0.56 | 0.48 | -2.09; 0.98 |
| **Psychological domain** | | |  | | |  |  |  |  |  |  |
| Psychological wellbeing | Individual sports | | Reference group | | |  |  |  |  |  |  |
|  | Team sports | | 0.80 | 0.13 | -0.25; 1.85 | 0.67 | 0.21 | -0.38; 1.72 | 0.46 | 0.39 | -0.60; 1.52 |
|  | Individual as well as team sports | | 0.70 | 0.30 | -0.63; 2.03 | 0.81 | 0.23 | -0.51; 2.13 | -0.08 | 0.93 | -1.54; 1.41 |
| Moods and emotions | Individual sports | | Reference group | | |  |  |  |  |  |  |
|  | Team sports | | -0.04 | 0.95 | -1.26; 1.17 | -0.20 | 0.75 | -1.41; 1.02 | -0.16 | 0.80 | -1.39; 1.07 |
|  | Individual as well as team sports | | -0.59 | 0.45 | -2.13; 0.95 | -0.46 | 0.56 | -1.99; 1.07 | -0.30 | 0.73 | -2.02; 1.41 |
| Self-perception | Individual sports | | Reference group | | |  |  |  |  |  |  |
|  | Team sports | | -0.14 | 0.81 | -1.24; 0.96 | -0.61 | 0.26 | -1.68; 0.46 | -0.61 | 0.27 | -1.69; 0.47 |
|  | Individual as well as team sports | | 0.24 | 0.74 | -1.16; 1.63 | 0.37 | 0.59 | -0.98; 1.71 | 0.39 | 0.61 | -1.11; 1.90 |
| **Social domain** |  | |  | | |  |  |  |  |  |  |
| Autonomy | Individual sports | | Reference group | | |  |  |  |  |  |  |
|  | Team sports | | 0.39 | 0.45 | -0.63; 1.41 | 0.23 | 0.66 | -0.79; 1.26 | 0.21 | 0.70 | -0.83; 1.24 |
|  | Individual as well as team sports | | -0.17 | 0.79 | -1.46; 1.12 | -0.09 | 0.89 | -1.38; 1.19 | -0.21 | 0.78 | -1.65; 1.24 |
| Parents and homelife | Individual sports | | Reference group | | |  |  |  |  |  |  |
|  | Team sports | | 0.56 | 0.26 | -0.43; 1.56 | 0.53 | 0.30 | -0.46; 1.52 | 0.30 | 0.55 | -0.69; 1.30 |
|  | Individual as well as team sports | | 0.30 | 0.64 | -0.96; 1.55 | 0.44 | 0.49 | -0.80; 1.69 | -0.48 | 0.50 | -1.87; 0.92 |
| Social support and peers | Individual sports | | Reference group | | |  |  |  |  |  |  |
|  | Team sports | | 0.77 | 0.15 | -0.27; 1.82 | 0.80 | 0.13 | -0.25; 1.86 | 0.65 | 0.23 | -0.41; 1.72 |
|  | Individual as well as team sports | | 0.99 | 0.14 | -0.33; 2.32 | 1.08 | 0.11 | -0.24; 2.40 | 0.44 | 0.56 | -1.04; 1.93 |
| Social acceptance (bullying) | Individual sports | | Reference group | | |  |  |  |  |  |  |
|  | Team sports | | 0.82 | 0.18 | -0.38; 2.03 | 0.98 | 0.11 | -0.23; 2.19 | 0.99 | 0.11 | -0.24; 2.21 |
|  | Individual as well as team sports | | 0.81 | 0.30 | -0.71; 2.33 | 0.89 | 0.25 | -0.63; 2.41 | 0.91 | 0.30 | -0.79; 2.62 |
| School environment | Individual sports | | Reference group | | |  |  |  |  |  |  |
|  | Team sports | | -0.98 | 0.06 | -2.01; 0.05 | -0.59 | 0.26 | -1.62; 0.44 | -0.78 | 0.14 | -1.82; 0.26 |
|  | Individual as well as team sports | | 0.36 | 0.59 | -0.94; 1.67 | 0.41 | 0.54 | -0.89; 1.70 | -0.39 | 0.60 | -1.84; 1.06 |
| Financial resources | Individual sports | | Reference group | | |  |  |  |  |  |  |
|  | Team sports | | 0.55 | 0.29 | -0.46; 1.57 | 0.58 | 0.26 | -0.43; 1.60 | 0.35 | 0.51 | -0.68; 1.37 |
|  | Individual as well as team sports | | 0.70 | 0.29 | -0.59; 1.98 | 0.81 | 0.21 | -0.47; 2.09 | -0.18 | 0.81 | -1.61; 1.25 |

^a^Adjusted for gender, age, BMI, SES, and household composition; ^b^Adjusted for gender, age, BMI, SES, household composition and frequency of sports participation; ^c^Unstandardized regression coefficient; ^d^p-values in bold indicate statistical significance (p < 0.05)

Table S5. Crude and adjusted analyses of the associations between performing indoor versus outdoor sports and HRQOL-dimensions for sports club members (n=1,603)

|  |  | Crude analyses | | | Adjusted analyses^a^ | | | Adjusted analyses^b^ | | |
| --- | --- | --- | --- | --- | --- | --- | --- | --- | --- | --- |
|  |  | B^c^ | p^d^ | 95%CI | B^c^ | p^d^ | 95%CI | B^c^ | p^d^ | 95%CI |
| **Physical domain** | |  | | |  |  |  |  |  |  |
| Physical wellbeing | Indoor sports | Reference group | | |  |  |  |  |  |  |
|  | Outdoor sports | 2.80 | **<0.001** | 1.77; 3.82 | 1.81 | **0.001** | 0.73; 2.88 | 0.97 | *0.08* | -0.13; 2.06 |
|  | Indoor versus outdoor sports | 0.95 | 0.21 | -0.53; 2.44 | 1.13 | 0.13 | -0.32; 2.59 | -1.02 | 0.20 | -2.59; 0.55 |
| **Psychological domain** | |  | | |  |  |  |  |  |  |
| Psychological wellbeing | Indoor sports | Reference group | | |  |  |  |  |  |  |
|  | Outdoor sports | 1.33 | **0.01** | 0.36; 2.29 | 0.89 | *0.09* | -0.13; 1.92 | 0.54 | 0.31 | -0.51; 1.59 |
|  | Indoor versus outdoor sports | -0.23 | 0.75 | -1.62; 1.16 | -0.11 | 0.88 | -1.49; 1.27 | -1.02 | 0.19 | -2.53; 0.49 |
| Moods and emotions | Indoor sports | Reference group | | |  |  |  |  |  |  |
|  | Outdoor sports | 1.65 | **0.004** | 0.53; 2.76 | 1.31 | **0.03** | 0.12; 2.50 | 1.37 | **0.03** | 0.15; 2.59 |
|  | Indoor versus outdoor sports | -0.86 | 0.29 | -2.47; 0.74 | -0.71 | 0.39 | -2.31; 0.90 | -0.55 | 0.54 | -2.30; 1.20 |
| Self-perception | Indoor sports | Reference group | | |  |  |  |  |  |  |
|  | Outdoor sports | 2.22 | **<0.001** | 1.21; 3.22 | 0.98 | *0.07* | -0.06; 2.03 | 0.97 | *0.08* | -0.11; 2.04 |
|  | Indoor versus outdoor sports | 0.13 | 0.86 | -1.32; 1.58 | 0.32 | 0.66 | -1.10; 1.73 | 0.28 | 0.73 | -1.27; 1.82 |
| **Social domain** | |  | | |  |  |  |  |  |  |
| Autonomy | Indoor sports | Reference group | | |  |  |  |  |  |  |
|  | Outdoor sports | 1.05 | **0.03** | 0.13; 1.99 | 0.77 | 0.13 | -0.23; 1.77 | 0.70 | 0.18 | -0.32; 1.73 |
|  | Indoor versus outdoor sports | -0.91 | 0.19 | -2.26; 0.44 | -0.83 | 0.23 | -2.18; 0.52 | -1.01 | 0.18 | -2.48; 0.47 |
| Parents and home life | Indoor sports | Reference group | | |  |  |  |  |  |  |
|  | Outdoor sports | 0.73 | 0.12 | -0.18; 1.65 | 0.50 | 0.31 | -0.47; 1.47 | 0.16 | 0.76 | -0.83; 1.15 |
|  | Indoor versus outdoor sports | -0.34 | 0.61 | -1.65; 0.98 | -0.21 | 0.75 | -1.52; 1.10 | -1.10 | 0.13 | -2.53; 0.32 |
| Social support and peers | Indoor sports | Reference group | | |  |  |  |  |  |  |
|  | Outdoor sports | -0.01 | 0.98 | -0.97; 0.95 | -0.09 | 0.86 | -1.12; 0.94 | -0.42 | 0.44 | -1.47; 0.64 |
|  | Indoor versus outdoor sports | -0.50 | 0.48 | -1.89; 0.89 | -0.40 | 0.57 | -1.80; 0.99 | -1.25 | 0.11 | -2.76; 0.27 |
| Social acceptance (bullying) | Indoor sports | Reference group | | |  |  |  |  |  |  |
|  | Outdoor sports | 1.75 | **0.002** | 0.64; 2.86 | 2.15 | **<0.001** | 0.97; 3.33 | 2.16 | **<0.001** | 0.95; 3.38 |
|  | Indoor versus outdoor sports | 0.35 | 0.66 | -1.24; 1.95 | 0.44 | 0.59 | -1.15; 2.03 | 0.47 | 0.6 | -1.27; 2.20 |
| School environment | Indoor sports | Reference group | | |  |  |  |  |  |  |
|  | Outdoor sports | -1.52 | **0.002** | -2.48; -0.57 | -0.85 | 0.10 | -1.86; 0.16 | -1.21 | **0.02** | -2.24; -0.17 |
|  | Indoor versus outdoor sports | -0.20 | 0.78 | -1.56; 1.17 | -0.19 | 0.78 | -1.55; 1.17 | -1.12 | 0.14 | -2.60; 0.36 |
| Financial resources | Indoor sports | Reference group | | |  |  |  |  |  |  |
|  | Outdoor sports | 1.00 | **0.04** | 0.06; 1.93 | 0.93 | *0.07* | -0.06; 1.93 | 0.58 | 0.27 | -0.44; 1.60 |
|  | Indoor versus outdoor sports | 0.31 | 0.66 | -1.04; 1.65 | 0.41 | 0.55 | -0.94; 1.75 | -0.51 | 0.49 | -1.97; 0.95 |

^a^Adjusted for gender, age, BMI, SES, and household composition; ^b^Adjusted for gender, age, BMI, SES, household composition and frequency of sports participation; ^c^Unstandardized regression coefficient; ^d^p-values in bold indicate statistical significance (p < 0.05)
